# The “Seminartage Weiterbildung Allgemeinmedizin” (SemiWAM^®^) – development, implementation and evaluation of a five-year, competence-based postgraduate programme in Bavaria

**DOI:** 10.3205/zma001540

**Published:** 2022-04-14

**Authors:** Marco Roos, Antonius Schneider, Jochen Gensichen, Anne Simmenroth, Thomas Kühlein, Dagmar Schneider

**Affiliations:** 1 Universität Augsburg, Medizinische Fakultät, Allgemeinmedizin, Augsburg, Germany; 2 Technische Universität München, Institut für Allgemeinmedizin und Versorgungsforschung, München, Germany; 3 Ludwig-Maximilians-Universität München, Institut für Allgemeinmedizin, München, Germany; 4 Universitätsklinikum Würzburg, Institut für Allgemeinmedizin, Würzburg, Germany; 5 Friedrich-Alexander-Universität Erlangen-Nürnberg, Allgemeinmedizinisches Institut, Erlangen, Germany; 6 Bavarian Competence Centre for Residency Training (BBCCRT), Munich, Germany

**Keywords:** curriculum, general practice, vocational training, postgraduate education, program evaluation

## Abstract

**Introduction:**

Starting in 2013, a five-year, competence-based postgraduate programme, the “Seminartage Weiterbildung Allgemeinmedizin” (SemiWAM^®^) for continuing education in general practice, was developed and offered in Bavaria. This evaluation reports on the experiences of SemiWAM^®^ after a first cycle.

**Material and methods:**

Process reflection based on the cycle of Kern: In addition to qualitative findings, results of the evaluation forms (mean values with standard deviation) are presented. The evaluation form contained questions on organisational issues, content of presentation, didactic preparation of the supervisor, transfer to real life practice as well as demographic variables. All questions were voted on a six-point Likert scale from “1=very satisfied” to “6=very dissatisfied”.

**Results:**

The reflection showed three crucial entry points: Choosing “reason for encounter” as a content precondition to ensure target audience needs, the close didactic supervision of supervisor, and the continuous growth of supervisor team with newly qualified GP. The evaluation results for the overall assessment (MW 1.11-1.60), the didactic concept (MW 1.30-1.87), as well as the transfer into daily life practice (MW 1.48-2.35) reflect the high quality of the SemiWAM^®^.

**Discussion:**

The SemiWAM^®^ curriculum presented can be easily transferred to comparable structures in Germany that accompany specialty training, such as the competence centres for residency training in general practice. The process evaluation based on the core cycle also provides important support for the agile implementation of these or similar programmes.

## Zusammenfassung

**Einleitung:**

Beginnend im Jahr 2013 wurde in Bayern ein fünfjähriges, kompetenzbasiertes, weiterbildungsbegleitendes Seminarcurriclum, die „Seminartage Weiterbildung Allgemeinmedizin“ (SemiWAM^®^) für die Weiterbildung in der Allgemeinmedizin entwickelt und angeboten. Mit diesem Evaluationsbericht werden die Erfahrungen der SemiWAM^®^ nach einem ersten Durchlaufen berichtet.

**Material und Methoden:**

Prozessreflexion auf Basis des Kern-Zyklus: Neben qualitativen Befunden werden Ergebnisse der Evaluation der Teilnehmenden (Mittelwerte mit Standardabweichung) dargestellt. Der Evaluationsbogen enthielt Fragen, u.a. zur Bewertung der Organisation, Inhalten und Art der Präsentation der Referierenden, der didaktischen Aufbereitung, des Transfers in die Weiterbildungspraxis sowie demographische Variablen. Alle Fragen wurden auf einer sechststufigen Likertskala von „1=sehr zufrieden“ bis „6=sehr unzufrieden“ abgestimmt.

**Ergebnisse:**

In der Prozessreflexion zeigten sich drei Stellschrauben als zielführend: Die Ausrichtung der Inhalte an Beratungsanlässen nah am Bedarf der Zielgruppe, die enge didaktische Supervision von Referierendenteams und die stetige Erweiterung des Referierendenteams durch junge Fachärztinnen und Fachärzte für Allgemeinmedizin. Die Evaluationsergebnisse für die Gesamtbewertung (MW 1,11-1,60), die didaktische Konzeption und Aufbereitung (MW 1,30-1,87) sowie den Transfer in die Weiterbildungspraxis (MW 1,48-2,35) spiegeln die hohe Qualität der SemiWAM^®^.

**Diskussion:**

Das vorgestellte Curriculum der SemiWAM^®^ lässt sich zwanglos auf vergleichbare weiterbildungsbegleitende Strukturen in Deutschland wie die Kompetenzzentren Weiterbildung Allgemeinmedizin übertragen. Die Prozessevaluation auf Basis des Kern-Zyklus gibt zudem wichtige Hilfestellung in der agilen Umsetzung dieser oder ähnlicher Programme.

## 1. Introduction

Medical education is increasingly oriented towards competency models such as the CanMEDS model, which is frequently used internationally [1]. In Germany, too, the aim is to align pre- and postgraduate training with this model [2], [3]. Due to shortage of general practitioners, structure and content of specialty training have been improved [4], [5], [6], [7]. The need for a competence-oriented curriculum for specialty training was taken up by the German Society for General Practice and Family Medicine (DEGAM) in 2014 and is available as a competence-based curriculum in general practice (CCGP) [8]. 

### 1.1. Specialty training in general practice in Germany

Although the 16 medical associations in each federal state of Germany have detailed regulations for specialty training, the training is structurally and educationally predominantly unorganized. Clinical rotations are usually neither attached to training programs nor to academic centres and have to be organized by the trainees themselves [9]. The advantages of this lack of structure are great degrees of freedom and highly individualized and flexible training. Trainees can change their field of specialisation at any time. Institutions benefit from a large number of trainees they can employ according to their needs [10], [11], [12]. In 2009, an international expert commission for specialty training in general practice confirmed that there is a need to catch the specialty training up to European standards with regard to structure, organisation, remuneration, and orientation towards competencies [13].

The “Versorgungsstärkungsgesetz” (an act to strengthen the provision of health care in Germany) introduced the establishment of “Competence Centres for Residency Training in General Practice” (CCRT) to increase efficiency and quality in specialty [14], [15]. This resulted in 14 CCRT that are funded according to section 75a SGB V [https://www.ge-weiterbildung.de/de/kompetenzzentren-weiterbildung.php]. 

#### 1.2. Specialty training in general medicine in Bavaria

The Bavarian Medical Association, the Association of Statutory Health Insurance Physicians of Bavaria, the Coordination Office for General Practice, the Bavarian Association of General Practitioners and the Chairs of General Practice at the Universities of Munich, Wuerzburg and Erlangen-Nuremberg have been cooperating in the Bavarian Competence Centre for Residency Training (BCCRT) since 2017. The obligatory tasks of the BCCRT include a structured seminar programme to accompany specialty training, a mentoring programme and a train-the-trainer program for training supervisors. For the first time, public financial support makes it possible to finance training structure beyond the employment relationship of trainees. In addition to some basic funding, the BCCRT receives a maximum annual amount of €750 for each trainee. In 2020, 434 trainees took part in the seminar program of the BCCRT.

#### 1.3. Aim 

In 2013, the Coordination Office for General Practice initiated the development of a seminar programme for general practitioners in Bavaria to accompany their specialty training. Since 2015, the seminar programme called “Seminartage Weiterbildung Allgemeinmedizin (SemiWAM^®^)” have been offered regularly, since 2017 as a BCCRT offer.This evaluation reports on the experiences of the development, implementation, and evaluation of the 5-year seminar curriculum of SemiWAM^®^.

## 2. Material and methods

Kern et al. provide a commonly used six-step framework for curriculum development, implementation, and evaluation [16]:

1. Problem identification.
2. Targeted needs assessment through a nominal expert panel consensus [17].
3. Development of goals and objectives: these were formulated into a first grid from the results of the expert panel consensus and the literature available for the German context in a development team (authors MR and DS) [15], [18], [19], [20], [21], [22]. The detailed elaboration took place in “ongoing operation” at each SemiWAM^®^.
4. Adaptation of methodology and didactics: For the purpose of uniform implementation of qualification measures and supervision structures [23], [24], regular retreat meetings of the trainers were introduced.
5. Implementation and analysis of hindering factors.
6. Evaluation of success: Levels 5 and 6 were obtained by observation at the individual SemiWAM^®^ and by means of standardised evaluation by participating trainees. The evaluation form, which changed slightly over the years, contained questions on the structural organisation, content of presentation, didactic preparation of the supervisor, as well as demographic variables. In addition, the transfer of the content into everyday practice was queried. All questions were rated on a six-point Likert scale from “1'=very satisfied” to “6=very dissatisfied”.

### Data processing

All qualitative data (stages 1-5) are based on protocols and participant observation of the development team (authors MR and DS) as well as the written documents of the curriculum and the implementation of the SemiWAM^®^. All reported quantitative evaluation data come from paper-based questionnaires. The results are presented purely descriptively. Calculations were made using Microsoft Excel (version Excel 2019, Microsoft Corporation, Redmond, USA). 

## 3. Results

### 3.1. Problem definition and targeted needs assessment 

A round table with representatives of the Bavarian Medical Association, the Association of Statutory Health Insurance Physicians of Bavaria, the Bavarian Association of General Practitioners, representatives of trainees and newly qualified doctors and the chairs of departments for general practice at the medical faculties in Munich and Erlangen analysed existing specialty training offers in a first step. Afterwards, an initial collection of topics for the SemiWAM^®^ was developed. A total of three working meetings took place (Q3/2014-Q2/2015). 

From the beginning, the preconditions for the SemiWAM^®^ were different compared to other usual medical training formats. The content of each seminar section follows a reason for encounter. The leading idea is to offer topics close to the needs of the GPs, and, in addition, to enable a direct transfer away from disease-based knowledge to clinical decision-making knowledge in daily practice. As a direct consequence the teaching should be carried out by general practitioners who are qualified to give lectures. If this standard cannot be met, at least a tandem of general practitioner and specialist physician must be formed. The SemiWAM^®^ should convey the general practitioner's attitude of evidence-based medicine, which combines individual patient-centredness, a critical appraisal of guidelines (especially those of the German College of General Practitioners and Family Physicians – DEGAM) and external evidence, as well as their own medical expertise. The presentation of conflicts of interest and free of pharma-sponsoring seminars resulted from this. 

#### 3.2. Areas of competence and learning objectives

As a result, the five-year SemiWAM^®^ curriculum was developed. The programme consists of four full-day seminars per year, which corresponds to eight teaching units (TU) of 45 minutes each, respectively 32 TU per year and 160 TU per five years. The contents of the SemiWAM^®^ were aligned with the CCGP of DEGAM. Each seminar was designed to be standalone so that the trainees are independent of actual training rotation (inpatient/outpatient) and time of duration of specialty training. An overview of the topics of the SemiWAM^®^ is shown in table 1 (Tab. 1).

Based on this rough grid, a detailed blueprint with learning objectives was developed in the conception of each individual SemiWAM^®^ by the coordinating team and the respective supervisors. Table 2 (Tab. 2) shows an example of a blueprint for the SemiWAM^®^ on the topic of “Abdominal pain as a reason for encounter”.

#### 3.3. Methodology and didactics

Since 2015, regular retreat meetings (duration 2.5 days) of the supervisors have taken place, which served to qualify and reflect on the role as “supervisors” as well as to prepare the content and didactic methods of each SemiWAM^®^. The retreat meetings were used to plan the four SemiWAM^®^ for the following year. For each seminar, a group of supervisors was formed who developed the respective blueprints under the supervision of the coordinating team. These blueprints were peer-reviewed by the remaining supervisors and the coordinating team. Differences in the medical-didactic qualifications of the supervisors were levelled out and impulses for methods of didactic design were further developed. The members of the coordinating team are qualified through medical-didactic training (MME, didactic certificates or others). 

#### 3.4. Implementation and evaluation

Since 2015, the SemiWAM^®^ have been offered as a full-day seminar four times a year. For the implementation of the early SemiWAM^®^, the coordinating team relied on experienced supervisors from university (professors, teachers). Initially, groups of up to 40 trainees were accepted. Due to positive word-of-mouth advertising, many seminars were quickly overbooked and had to be offered several times.

From the second year onwards, the concept was changed to a seminar character with a maximum of 25 trainees. This led to two crucial changes: It was necessary to switch to premises that offered both plenary sessions with up to 75 participants and sufficient seminar approaches for groups of 25 trainees each. In addition, there was a much greater need for supervisors. The frequent repetitions at different venues (Munich, Nuremberg, later Wuerzburg and Regensburg) could no longer be covered by the experienced supervisors. The coordinating team therefore decided to form teams of supervisors for each topic and to support them closely in terms of didactics. In addition to experienced supervisors, newly qualified general practitioners were recruited. They should serve as role models close to the life situation of the trainees. To support development of their role identification as a teacher, which had been less developed until then, close supervision by the coordinating team and detailed blueprint of each seminar were given. Additionally, for each seminar a team of experienced and young supervisors was formed. Since the introduction of the SemiWAM^®^ an annual increase in the number of participants has been achieved. Currently, the full-day seminars per topic are offered in Munich (for up to 6x25 trainees), Nuremberg (up to 3x25 trainees), Wuerzburg (up to 2x25 trainees) and Regensburg (up to 3x25 trainees). 

The evaluation results show a high degree of consent and satisfaction over the five years in the regularly surveyed questions, irrespective of the topics and supervisors. Likewise, there was narrow variation in the evaluation results within supervisors for each individual seminar day. The mean values for the overall evaluation are close together for the 20 seminar days (1.11-1.60). Likewise, the approval ratings for the didactic preparation (of 10) full-day seminars are at a high level (1.30-1.87). The greatest spread can be seen in the transfer of content into daily life practice, but it is also high (1.48 and 2.35) (see table 3 (Tab. 3)). 

## 4. Discussion

This article presents a longitudinal evaluation of a seminar curriculum for specialty training in General Practice. Based on the cycle by Kern et al., it was possible to reflect on important stages in the development and implementation. The five-year SemiWAM^®^ curriculum offers trainees the opportunity to acquire the content required for practice, in addition to specialty training in in- and outpatient rotations. The high concordance and satisfaction of trainees in the evaluation especially a high ranked transfer into daily practice reinforce this aspect. In addition, other aspects such as collegial exchange and identity building are fostered by the seminar [25].

Since an expert report on specialty training in general practice commissioned by DEGAM in 2009, important improvements have been achieved [13]. In the recent years, a competence-based curriculum (CCGP) was developed by a national working group of DEGAM [8], [20]. Constructive alignment and a didactic structure were initially lacking. In parallel to the developments in the specialty training scheme in Baden-Wuerttemberg, the Bavarian concept of SemiWAM^®^ was developed starting in 2014 [26], [27]. In contrast to already known topics for specialty training in general practice, the SemiWAM^®^ curriculum is characterised by its longitudinal coverage of all areas of the CCGP, which was iteratively improved during ongoing operation. This enabled an agile didactic adaptation based on the experience of past seminars on the one hand, and on the other hand, a quick reaction to a rapidly increasing response among the trainees. 

The process reflection proved three points in the implementation process to be purposeful: The content of seminars is oriented toward GP reasons for encounter and thus linked to daily training practice of the GPs. The close didactic supervision of supervisors on the one hand, and on the other hand, the constant increase of supervisors by newly qualified GP, who are experienced in daily practice. The process of developing topics for the 20 seminars in the SemiWAM^®^ curriculum turned out to be relatively simple. Due to the great breadth of the expert panel with participants from all areas of specialist training in general practice in Bavaria and the submission by the CCGP of DEGAM, the core competences of GP work were quickly identified. Such developments had also been carried out in parallel elsewhere and showed a high degree of congruence [4], [5], [7], [8], [18], [19], [20], [21], [22]. In our view, the orientation of the contents toward GP reasons for encounter represents an important factor for covering the needs of the target group. This was shown, for example, in the SemiWAM^®^ on “abdominal pain as a reason for encounter”. Abdominal pain is a symptom with many possible differential diagnoses from different disciplines. The implementation showed the importance of not focusing on possible diseases, but on prioritising management in the consultation. For this purpose, it was indispensable to use supervisors who work as GPs and who, in addition to their didactic role, also bring authentic experience from GP practice [28]. The high evaluation results and the confirmed transfer into daily practice by the participants impressively describe the reflective running through of the cycle of Kern et al. In addition, during the implementation of the first seminars, it was possible to fall back on academic and experienced supervisors. However, a conceptual expansion had to take place quickly. With the introduction of blueprints, the supervisors were set narrow didactic barriers in the design of the seminar. The blueprints describe the most important learning objectives and make suggestions for didactic implementation, adapting them to seminar groups of 25 trainees. This measure represented an important milestone in the quality assurance of the programme. In addition to the development of the content and didactics of the SemiWAM^®^ curriculum, there was a need for accompanying development and qualification of the supervisors. The coordinating team decided early on to recruit newly qualified GP who had already completed the SemiWAM^®^ as “alumni”. The direct proximity to the needs of the target group and the “socialisation” in the didactic framework of the SemiWAM^®^ spoke in favour. The very good evaluation results of the young supervisors confirm this approach and support the identification potential of “near peer” tutors described in the literature [29]. Close supervision by the coordinating team was also introduced here, which supported the role identification of the supervisors as “teachers”. The retreat meetings with developing the blueprints of each seminar and “developing” the supervisors were an important step to raise quality. 

The high evaluation scores of the content and the didactic methodology of each seminar days confirmed the current concept. In addition to the internal evaluation, the concept is also confirmed in the external evaluation of the National Association of Statutory Health Insurance Physicians [30]. This also supports the recruiting of young supervisors. The high values in the self-assessment of the transfer of the contents into daily practice is an important indication of the effectiveness of the curriculum beyond the reaction to the event [24]. This has already been confirmed in a controlled study in the important development of competence in dealing with diagnostic uncertainty [31].

### 4.1. Strengths and weaknesses 

The presented concept of a five-year seminar curriculum for specialty training in general practice can be transferred easily to other contexts in Germany, especially to structures such as the competence centres for specialty training in general practice according to §75 a SGB V. Two aspects appear to be promising in this context: on the one hand, central management with close supervision of content and didactics of the seminars, even with a high number of participants. On the other hand, the qualification of "near peer" supervisors under close supervision seems essential. A best practice model of interlinked curricular and personnel development is useful [32]. A further advantage also results from the type of programme. Trainees may attend the SemiWAM^®^ regardless of their actual training situation (outpatient or inpatient) or the duration of their training.

Limitations also emerged in the ongoing process. Although the current curriculum covers the core of GP work, the participating trainees mention other topics and needs in the free texts of the evaluation form, such as naturopathic treatment. Another disadvantage is the content preparing trainees for GP practice, which is considered less helpful for some trainees in clinical rotations. However, it is appreciated that the SemiWAM^®^ provides an overview of consultation practice and thus more psycho-emotional conviction for further training [33]. A general weakness lies in the voluntary nature of the offer. Up to now, the specialty training of health professionals in Germany has not been tied to central registration or to mandatory training schemes. A large proportion of trainees in Bavaria do not use the SemiWAM^®^ in their specialty training, but join intermediate: a repeated curriculum every 2-3 years would therefore be useful. Up to now, it has been more important to the organisers to explicitly establish a five-year curriculum parallel to the regulations of specialty training. A selection bias among the participating trainees cannot be ruled out. However, with over 400 participating trainees in 2020, about a quarter of all financially supported trainees in Bavaria are involved. 

The methodological strength of this evaluation lies in the continuous evaluation with a fixed core of evaluation questions and the high response rate. A survey of supervisors was not the subject of this evaluation and should be supplemented in further studies. The effects after passing through the seminar curriculum could not be ascertained so far, but indications of the benefits were positively confirmed in parallel studies [31], [33].

#### 4.2. Next steps

In accordance with the cycle of Kern et al., it is now necessary to compare the needs with the own goals/evaluation results. In addition, due to the SARS-CoV-2 pandemic in 2020, it was necessary to react flexibly and switch to a web-based format, both for offering the curriculum and in the support of supervisors. Thus, since March 2020, all seminars have been didactically developed for online-based and face-to-face teaching. This requires further instruction for supervisors. For this purpose, regular online-based meetings have already been introduced for the reflection of the supervisors. Since 2020, individual contents of the SemiWAM^®^ have been offered beyond the basic curriculum. These are partly specific to individual contexts (such as short advanced training on the S1 guideline “SARS-CoV-2”) or developed at the request of trainees (12-week module on practice management). Another goal is to link further offers of the BCCRT like train-the-trainer or mentoring to the SemiWAM^®^. Seminars for trainer in specialised training organisations that reflect the contents of the curriculum appear to be goal oriented. In addition, an evaluation of suitable aids to support the trainees in transferring the contents into daily training practice should be expanded.

## 5. Conclusion

The SemiWAM^®^ are an example of a five-year, competence-based seminar programme accompanying specialty training that covers all the core competences defined in the DEGAM's competence-based curriculum. The curriculum presented can be easily transferred to comparable specialty training structures in Germany, such as further competence centres for specialty training in general practice. The evaluation provides important support for the agile implementation of such or similar programmes. 

## Funding

Until June 2017, the SemiWAM^®^ were financially supported by the Bavarian State Ministry of Health and Care. Since 2017, the SemiWAM^®^ have been carried out within the framework of the Competence Centre for Residency Training in General Practice in Bavaria (BCCRT) by the Coordination Office for General Medicine Bavaria (KoStA) and are financially supported within the legal regulations of §75 a SGB V. 

## Acknowledgement

The authors would like to thank Ulrike Seider, Cornelia Dodeller, Yvonne May, and Markus Degner for organising the SemiWAM^®^ and all the supervisors: Tom Brandhuber, Wolfgang Blank, Jessica Bungartz-Catak, Johanna Burgis, Michael Diemer, Anna Frangoulis, Elena Fuchs, Susanne Heydner, Charlotte Hoser, Johanna Jais, Berthold Jeßberger, Ronny Jung, Barbara Kaiser-Marner, Reinhold Klein, Martin Kotowicz, Raphael Kunisch, Claudia Levin, Elisabeth Rieck, Michael Rinecker, Stefan Roi, Matthias Schaller, Angela Schedlbauer, Jörg Schelling, Lothar Schmittdiel, Miriam Schrön, Esther Seifert, Melanie Tretter, Katja Tritzschler, Hanna Unger, Peter Wapler, Birgitt Weinhold, and Peter Zehentner.

## Competing interests

The authors declare that they have no competing interests. 

## Figures and Tables

**Table 1:**
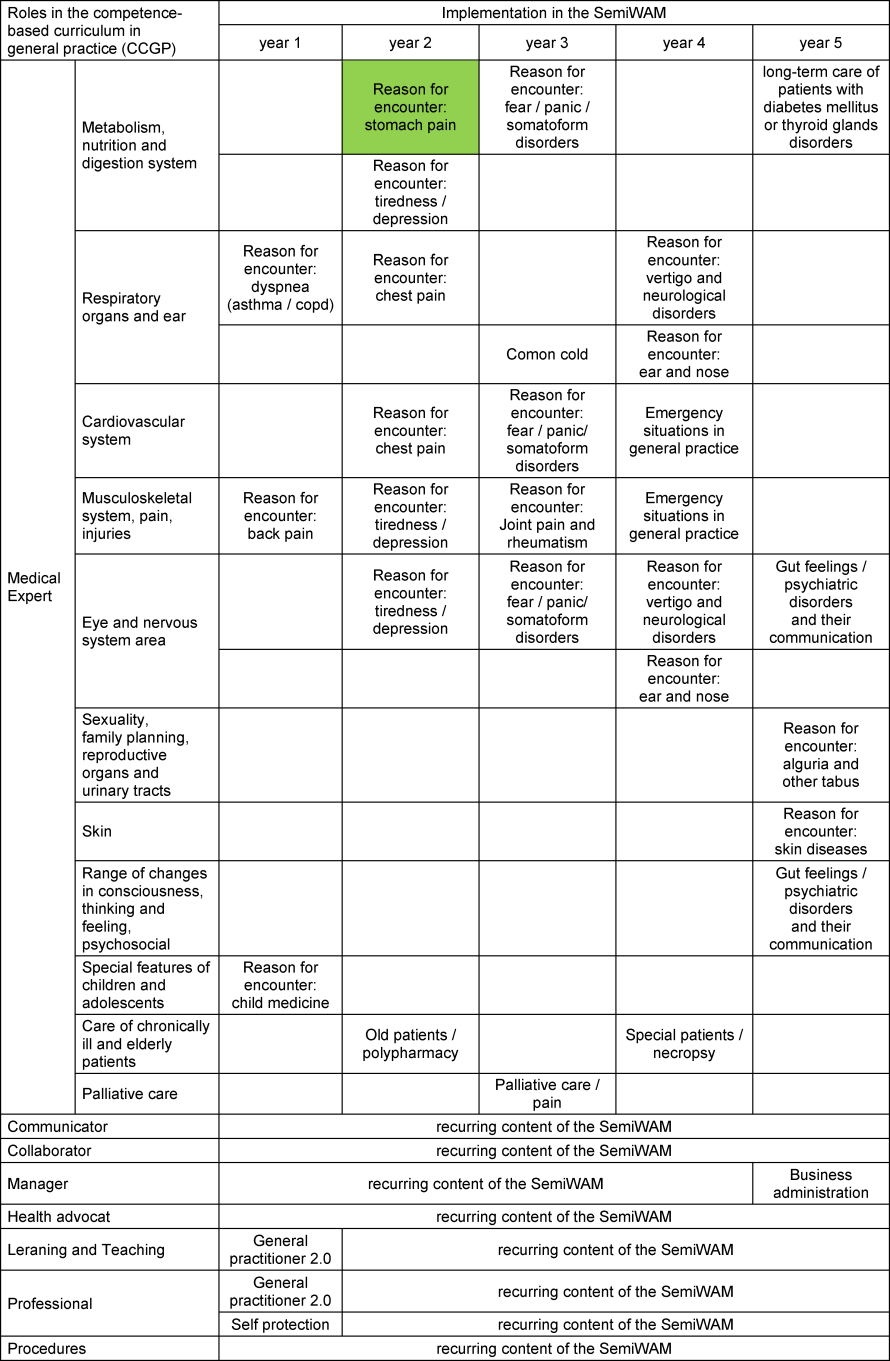
5-year curriculm of the SemiWAM^®^

**Table 2:**
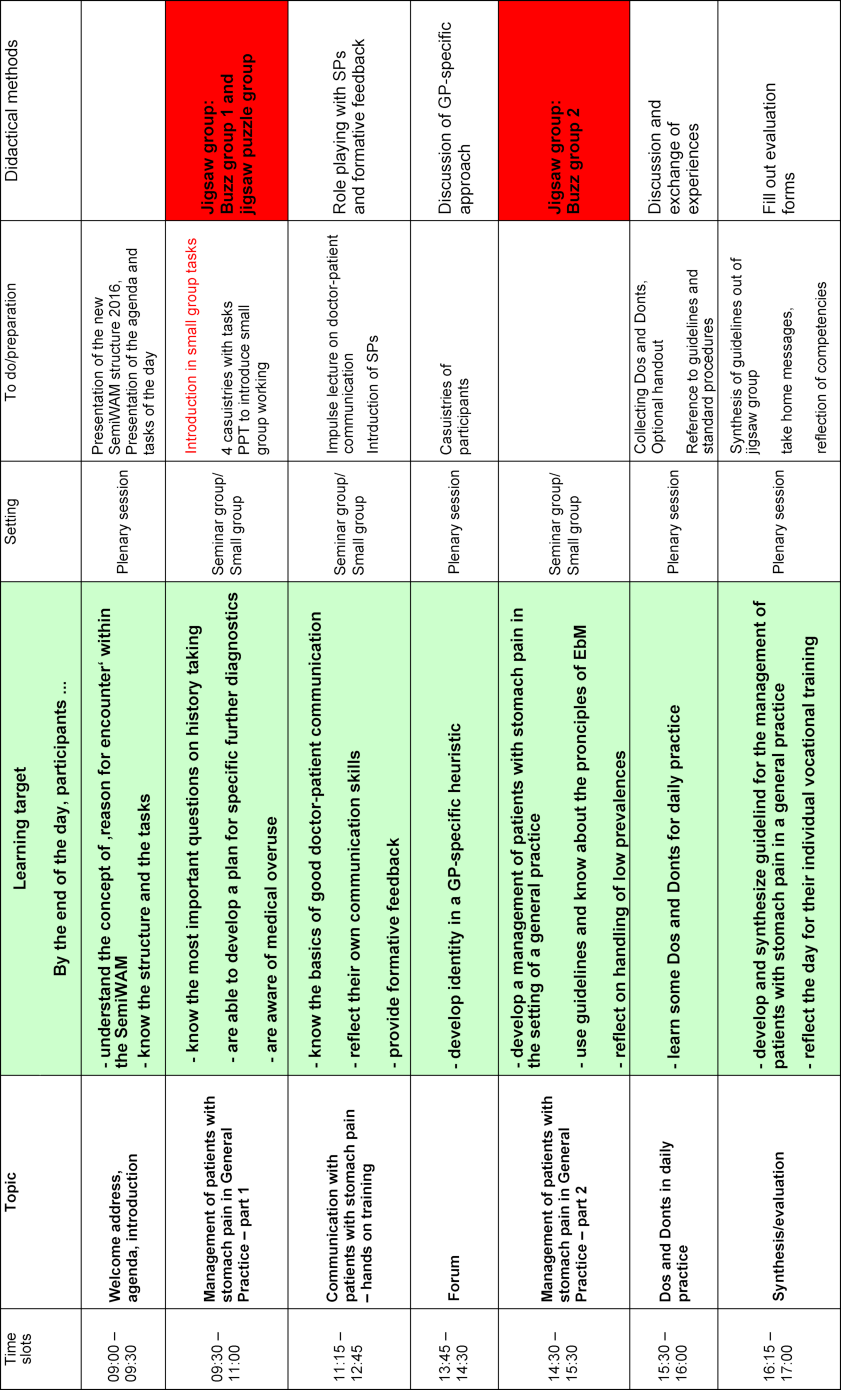
Blueprint – SemiWAM^®^ stomach pain

**Table 3:**
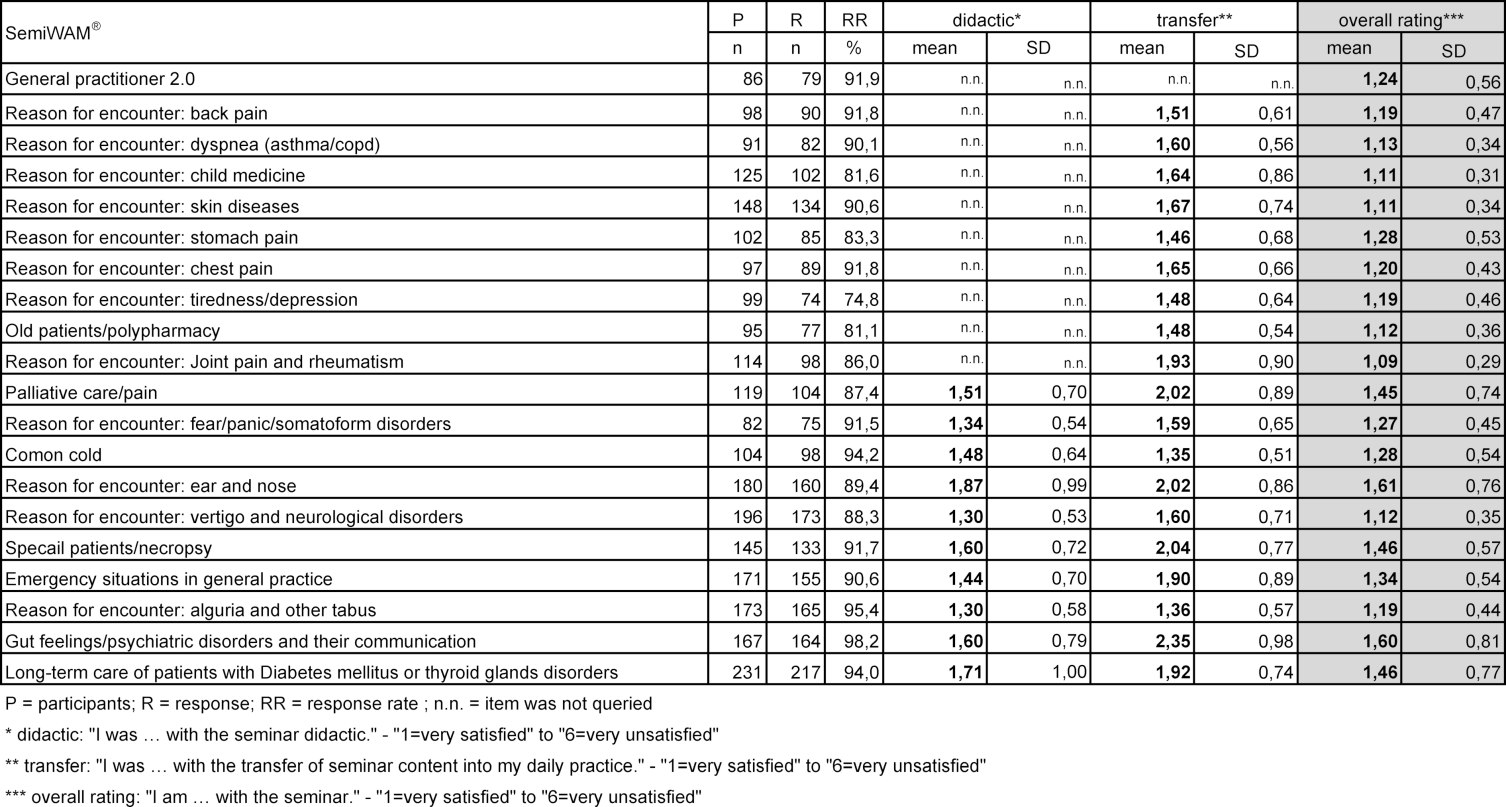
Evaluation results
